# Involvement in Health Behavior After Heart Transplantation: The Role of Personal Resources and Health Status. Single-Center Observational Study

**DOI:** 10.3389/fpsyg.2021.710870

**Published:** 2021-12-23

**Authors:** Anna Mierzyńska, Andrzej Kokoszka, Grażyna Jerzak-Wodzyńska, Małgorzata Sobieszczańska-Małek, Tomasz Zieliński, Ryszard Piotrowicz

**Affiliations:** ^1^Department of Coronary Artery Disease and Cardiac Rehabilitation, National Institute of Cardiology, Warsaw, Poland; ^2^Department of Cardiac Surgery, Military Institute of Medicine, Warsaw, Poland; ^3^II Department of Psychiatry, Medical University of Warsaw, Warsaw, Poland; ^4^Department of Heart Failure and Transplantology, National Institute of Cardiology, Warsaw, Poland

**Keywords:** heart transplantation, adherence, health behavior, health practices, personal resources, personality, conscientiousness, health locus of control

## Abstract

**Introduction:** Heart transplantation affects all spheres of the patients’ functioning - their physical well-being and coping with everyday situations, as well as their identity and social functioning. Its long-term effects depend on the effective cooperation with the transplant team. Post-transplant patients are expected to be committed to adherence to recommendations. Patients’ subjective characteristics could increase the risk of difficulties during treatment or might have a protective effect. The major aim of the study was to evaluate the level of engagement in health behavior in heart transplant recipients in relation to their personal resources, such as personality traits, and their health status.

**Material and Method:** The observational *ex post* facto model was proposed. Participants completed a set of psychological questionnaires. In the study, there were used questionnaires regarding health behavior (HBI), personality traits (NEO-FFI), health locus of control (MHLC), self-efficacy (GSES) and health status (GHQ-28). The group included in the analyses consisted of 107 heart transplant patients. They ranged in age from 19 to 75 years; 10.3% of them were women.

**Results:** According to norms, 71% patients reported high level of engagement in health behavior. There were significant differences in the level of dietary habits and other types of health behaviors. The best predictors of overall health behavior were conscientiousness (β = 0.20, *p* < 0.05) and health locus of control (Powerful Others) (β = 0.25, *p* < 0.05). The prophylaxis behavior was related significantly to the level of conscientiousness (*p* < 0.05) and health locus of control (Internal and Powerful Others) (*p* < 0.05; *p* < 0.01). The level of positive mental attitude was related significantly to agreeableness (*p* < 0.05), health locus of control (Powerful Others) (*p* < 0.01), and self-efficacy (*p* < 0.01). Everyday healthy practices were related significantly to openness to experience (*p* < 0.01) and health locus of control (all categories: Internal, Powerful Others and Chance) (*p* < 0.05; *p* < 0.01; *p* < 0.05, respectively).

**Conclusion:** Majority of heart transplant patients is engaged in high level of health behavior. Among the various forms of health-relevant habits, heart transplant patients adhere significantly less frequently to a healthy diet. Among examined resources, the best predictors of caring about health are generalized self-efficacy and age at the time of HTx.

## Introduction

Heart transplantation is an established method of treating end-stage heart failure, allowing it to extend the lives of people for whom other treatments have failed. It is a turning point event during heart failure treatment, and it affects all spheres of the patients’ functioning - their physical well-being and coping with everyday situations, as well as their emotional state, identity, and social functioning (Christopherson, 1987; Grady et al., 1999; Steinbüchel et al., 2000; Stolf and Sadala, 2006; Kugler et al., 2009; Ratajska, 2011; Mauthner et al., 2012, 2014; Pietruszewski and Siwy-Hudowska, 2013; Abedi et al., 2015). In recent years, about 80-100 procedures have been performed annually in Poland, and their number has remained constant for about 8 years (Malanowski, 2018). Despite the recent introduction of innovative technologies to support the left ventricle at home (LVAD), heart transplantation remains the most frequently chosen method of treating advanced heart failure (Cadeiras et al., 2007). Heart transplantation is a treatment method whose long-term effects depend on the effective cooperation of patients with the treatment team. The care regimen after heart transplantation includes frequent outpatient control visits and invasive medical tests, such as biopsies or coronarography, especially in the first years after surgery, and patients are required to conscientiously take medications and follow medical recommendations relating to various spheres of everyday functioning (Zieliński and Sobieszczańska-Małek, 2015). Treatment after heart transplantation places new demands on the patient and his or her immediate environment. The most important tasks of the patient, analogously to the situation of treatment of other somatic diseases, include coping with the psychological consequences of their condition and treatment, adapting to the requirements of treatment (especially immunosuppressive) and the specific environment, which is the health service, and building and maintaining a positive relationship with the treatment team (Kubacka-Jasiecka and Ziarko, 2016). Post-transplant patients are expected to be committed to adherence to recommendations (understood primarily as scrupulously taking prescribed medications) and to adapt their lifestyles to the principles of treatment, i.e., taking care of their general health, avoiding risks in the form of infections (Barańska-Kosakowska et al., 2013; Dębska-Ślizień et al., 2015). This is to enable patients to prolong their lives and to minimize the risk of complications such as vasculopathy or new organ rejection (Sobieszczańska-Małek, 2015).

The literature indicates the influence of several groups of factors on the potential for cooperation after organ transplantation affecting patients’ ability to follow recommendations and adhere to treatment. Those factors stem from the characteristics of the treatment and illness itself, organization of health-care system and patients’ personal and interpersonal resources. One of known major factors contributing to involvement in self-care is personality. Certain subjective characteristics of patients increase the risk of difficulties in coping with the demands of treatment. At the same time, personality traits are also known to have a protective effect. They influence patients to undertake behaviors consistent with health recommendations, which can be a valuable resource in the process of adaptation to treatment after heart transplantation. Findings from numerous studies suggest significant effects of individual personality traits on both overall health and lifestyle, as well as behaviors in the patient role, such as seeking medical help, coping with symptoms, and adherence to medical recommendations (Courneya and Hellsten, 1998; Widiger, 2005; Czarnecka and Tylka, 2010; Cheng et al., 2015; Čukiń et al., 2016; Kohli, 2017; Abbeya et al., 2018; Gray and Pinchot, 2018; Santangelo et al., 2018). Each of the personality traits exerts an individual influence on the individual, modifying the actions taken by him in different situational contexts, and the impact of the links of personality traits is synergistic in nature. Current studies regarding psychological factors influencing health behavior and compliance rarely refer to posttransplant patients. Considering that the role of good adjustment and adherence could be crucial to patients’ health status, quality of life, and survival, this knowledge could be beneficial in terms of patients care. Understanding the relationship between the psychological dispositions of heart transplant patients and their lifestyle and engagement in health-relevant behaviors may allow us to profile psychological factors important for cooperation related to their subjective characteristics. Therefore, the main research question in this study was whether stable psychological factors (such as personality traits) were important for sustaining healthy habits after heart transplantation. The major aim of the study was to evaluate the level of engagement in health behavior in heart transplant recipients in relation to their personal resources, such as personality traits, and their health status regarding somatic and psychological symptoms.

## Materials and Methods

In the study, the observational *ex post* facto model of the exploratory variety (implemented in the correlational mode) was proposed. Recruitment for the study took place at the Department of Heart Failure and Transplantation, Institute of Cardiology, Warsaw, Poland. The study was conducted within the framework of the statutory project no. 2.55/VII/12, the data collection for the cross-sectional part of the study was gathered from 2012 to 2014. The study received a positive opinion of the Bioethics Committee of the Institute of Cardiology (No. 1315).

Study participants were asked to complete a set of self-descriptive tools (psychological questionnaires) during hospitalization for medical follow-up examinations (e.g., cardiac tests and biopsy or other screening tests required by their current health status). The hospitalization period was chosen due to time-consuming self-evaluation using questionnaires and the need to give patients feedback. The self-assessment was conducted by the patients themselves with the presence of psychologist in case of any questions regarding items in questionnaires. Every patient was evaluated once during the study. At the conclusion of the study, participants received feedback regarding the level of their reported health behaviors in relation to norms, as well as assessments of psychological well-being and individual resources. Those who reported difficulties in functioning or adherence during the study received support according to the standards adopted by the transplant center.

In the study, there were used questionnaires regarding health behavior (HBI, Health Behavior Inventory by Z. Juczyński), personality traits (NEO-FFI, Five Factor Inventory by Costa and McCrae), health locus of control (MHLC, Multidimensional Health Locus of Control scale by Walston and Walston), self-efficacy (GSES, Generalized Self-Efficacy Scale by R. Schwarzer) and health status (GHQ-28, General Health Questionnaire by Goldberg) (Zawadzki et al., 1998; Goldberg et al., 2001; Juczyński, 2010). The HBI scale consists of 28 items regarding various forms of health behavior, and it allows to describe subject’s health behavior pattern related to 4 areas: healthy dietary habits (HDH), prophylaxis behavior (PB), positive mental attitude (PMA) and everyday healthy practices (EHP), as well as the general health behavior index (HBI). The higher result of the HBI and its subscales indicates the higher level of particular behavior. The score range for HBI is 24–120 points, and for every area – from 1 to 5 points based on the calculator proposed by the Authors. The general score could be transformed to sten scale according to norms for gender (Juczyński, 2010). The NEO-FFI refers to the OCEAN personality model (Neuroticism, Extraversion, Openness, Agreeableness, Consciousness) and the score range for every scale is between 12 and 60 points (Zawadzki et al., 1998). The MHLC questionnaire evaluates the health locus of control in 3 dimensions (Internal, External – Powerful Others, and External – Chance) with a score range of 6–36 points for every scale (Juczyński, 2010). The GSES scale refers to the generalized self-efficacy concept and its score ranges from to 10 to 40 points. The GHQ-28 scale results were calculated with the method assuming 0–3 points for every item, according to the method recommended by the Authors. The score for every subscale (somatic symptoms, anxiety with insomnia, social dysfunction, and depression) ranges from 0 to 21 points, with the general score (overall distress) in the range of 0–48 points. The overall GHQ-28 score could be transformed to sten scale according to norms for gender (Goldberg et al., 2001).

### Study Group

The study was conducted according to analytical observational design and the research model assumed the use of a non-randomized study sample. Data collection used a purposive sampling method, according to the focus of the study and the population characteristics sought (Beins and McCarthy, 2018). The inclusion criteria for the study were being adult, more than 1 year after heart transplantation, and treatment at the transplant center of the Institute of Cardiology in Warsaw, and exclusion criteria consisted of having severe cognitive impairment or health status disabling the use of self-assessment, or withdrawal of consent during the study. At the time of participating in the study patients had stable health status, with a satisfactory level of functioning. The reason of not recruiting patients who were less than 1 year post-transplant was that it is a time of early adjustment to a new situation and medical regimen, with a higher risk of early post-surgery complications. The study aimed at the group of assumed stable health behavior patterns in post-transplant care regarding patients’ individual resources.

After collecting quantitative data from 118 subjects, data from 107 subjects were used for further analyses. The reason for exclusion from the analysis was improper completion of the self-report questionnaires (numerous missing answers or selection of several answers to a given question), which prevented reliable analysis of the full set of questionnaires.

The group included in the analyses consisted of 107 heart transplant patients. They ranged in age from 19 to 75 years; 10.3% of them were women. The time since transplantation of the subjects was 1 year to 24 years at the time of inclusion in the study. Majority of the subjects had a high school education. A detailed summary of the demographic characteristics of the study subjects is shown in Table 1.

**TABLE 1:** Characteristics of the subjects participating in the study.

	Number of participants (*N* = 107)
Gender	11 females (10.3% of the group)
	96 males (89.7% of the group)
Age	*M* = 53.11; SD = 14.39 Min = 19/Max = 75
Age at time of heart transplantation	*M* = 46.36; SD = 13.37 Min = 14/Max = 66
Time since heart transplantation	*M* = 6.75; SD = 4.76 Min = 1/Max = 24
Education	
Primary education	8 patients (7.5% of the group)
Occupational education	31 patients (37% of the group)
Secondary education	50 patients (46.7% of the group)
Higher education	15 patients (14% of the group)
No data	3 patients (2.8% of the group)

Patients most often reported some form of cardiomyopathy as the main reason for qualifying for heart transplantation (59 people, 23 of whom did not specify the type). Among the study participants who were able to name the type of cardiomyopathy, 22 named dilated cardiomyopathy and 5 named hypertrophic cardiomyopathy. The next most common reason for qualification for transplantation was myocardial infarction and its consequences (33 people, 30.84% of the group). The remaining patients reported other reasons, such as postinfectious complications, valvular defects, or arrhythmogenic cardiomyopathy.

### Statistical Analysis

The results of the study were compiled using the statistical analysis program SPSS.20 (OS: Win 10). Descriptive results for nominal variables are in the form of absolute numbers and percentages for the entire study group. The results for continuous variables are presented as means and standard deviations. The distribution of variables was assessed using Shapiro-Wilk method. The differences in distinct types of health behaviors were examined using Wilcoxon rank test. To verify hypotheses on the influence of single factors on the examined variables, Spearman’s rank correlation method (for variables of non-normal distribution) or Pearson’s linear correlation (for variables of normal distribution) were used. The difference in health behavior in groups with lower and higher levels of education was analyzed using the U Mann-Whitney test (the first group consisted of patients with primary and occupational education, which means less than 12 years of education, and the second group consisted of participants with secondary and higher education – 12 and more years of education). The next step of the analyzes was to determine the interaction of predictors on the dependent variables by running a multiple regression model including the stepwise method of introducing the predictors one at a time. A significance level of *p* < 0.05 was assumed for reporting statistically significant results.

## Results

Based on the collected data, the level of engagement in health behaviors in the group of heart transplant recipients was determined. Descriptive statistics of health behaviors (overall index and individual types of health behaviors) and psychological variables are presented in Tables 2, 3. According to Polish norms 71% patients reported a high level of engagement in health behavior, whereas 24.3% declared average and 4.7% – low level of health behavior (Juczyński, 2010). There were significant differences in the level of dietary habits and other types of health behavior, with patients being less engaged in dietary behaviors in comparison to prophylaxis, positive mental attitude, and daily healthy practices (Table 4).

**TABLE 2:** Descriptive statistics of individual types of health behaviors.

Descriptive statistics (*N* = 107)	Mean (M)	Standard deviation (SD)	Minimum (Min)	Maximum (Max)
HBI	92.92	11.90	56	118
HDH	3.56	0.73	1.50	5.00
PB	4.01	0.66	1.67	5.00
PMA	3.98	0.54	2.67	5.00
EHP	3.94	0.61	2.17	5.00

*HBI – heath behavior index; HDH – healthy dietary habits; PB – prophylaxis behavior; PMA – positive mental attitude; EHP – everyday healthy practices.*

**TABLE 3:** Descriptive statistics of psychological variables.

Descriptive statistics (*N* = 107)	Mean (M)	Standard deviation (SD)	Minimum (Min)	Maximum (Max)
NEU	18.45	5.82	6	34
EXT	26.47	4.58	17	42
OPN	24.69	5.10	13	40
AGR	30.10	4.97	14	39
CON	32.64	5.39	15	45
HLoC-In	24.70	5.43	12	36
HLoC-PO	28.05	4.59	17	36
HLoC-Ch	21.07	6.27	8	36
GSE	31.30	3.85	20	40
GHQ-28 Som	6.70	3.98	1	17
GHQ-28 Anx	5.58	3.70	0	16
GHQ-28 Soc	7.41	3.28	0	19
GHQ-28 Depr	1.88	2.29	0	10
GHQ-28 Diss	21.57	10.16	4	50

*HTx – heart transplantation; HBI – heath behavior index; HDH – healthy dietary habits; PB – prophylaxis behavior; PMA – positive mental attitude; EHP – everyday healthy practices; NEU – Neuroticism; EXT – Extraversion; OPN – Openness to experience; AGR – Agreeableness. CON – Conscientiousness. HLoC-In – Health Locus of Control – Internal; HLoC-PO – Health Locus of Control – Powerful Others; HLoC-Ch – Health Locus of Control – Chance; GSE – Generalized Self-Efficacy; GHQ-28 Som – Somatic Symptoms; GHQ-28 Anx – Anxiety with Insomnia; GHQ-28 Soc – Social Disfunction; GHQ-28 Dep – Depression; GHQ-28 Diss – Overall Distress.*

**TABLE 4:** Differences between types of health behaviors.

	PB – HDH	PMA – HDH	EHP – HDH	PMA – PB	EHP –PB	EHP – PMA
Z	–5.91	–5.54	–4.66	–1.00	–1.00	–0.,60
Significance (two-tailed)	0.00*	0.00*	0.00*	0.32	0.32	0.55

*p < 0.001.

*HBI – heath behavior index; HDH – healthy dietary habits; PB – prophylaxis behavior; PMA – positive mental attitude; EHP – everyday healthy practices.*

Among the examined relationships, the correlation between the level of conscientiousness and the level of overall health behaviors (HBI) reached the required level of statistical significance (*r* = 0.23; *p* < 0.01). Furthermore, the level of statistical significance was obtained by the relationships concerning self-efficacy (*r* = 0.17, *p* < 0.05) and health locus of control (all categories: Internal, Powerful Others and Chance) (*r* = 0.23, *p* < 0.01; *r* = 0.27, *p* < 0.01; *r* = 0.17, *p* < 0.05, respectively). Since all correlation coefficients are at a level below 0.3, the strength of the relationship between these variables should be described as weak. The remaining correlations with HBI did not reach the required level of statistical significance. There was also relationship between personality factors and three of four types of health behavior observed. The prophylaxis behavior was related significantly to the level of conscientiousness (*r* = 0.22, *p* < 0.05) and health locus of control (Internal and Powerful Others) (*r* = 0.21, *p* < 0.05; *r* = 0.29, *p* < 0.01). The level of positive mental attitude was related significantly to agreeableness (*r* = 0.21, *p* < 0.05), health locus of control (Powerful Others) (*r* = 0.28, *p* < 0.01) and self-efficacy (*r* = 0.28, *p* < 0.01). Everyday healthy practices were related significantly to openness to experience (*r* = -0.23, *p* < 0.01) and health locus of control (all categories: Internal, Powerful Others and Chance) (*r* = 0.17, *p* < 0.05; *r* = 0.28, *p* < 0.01; *r* = 0.21, *p* < 0.05, respectively). The remaining correlations were non-significant (Table 5).

**TABLE 5:** Correlation between health behaviors, personality factors, health status and demographical factors.

		HBI	HDH	PB	PMA	EHP
NEU	r	–0.070	0.002	–0.083	–0.141	–0.036
	p	0.236	0.491	0.198	0.074	0.358
EXT	r	–0.086	–0.155	0.028	0.057	–0.138
	p	0.189	0.056	0.387	0.280	0.079
OPN	r	–0.068	0.067	–0.021	–0.030	–0.226**
	p	0.243	0.247	0.413	0.378	0.010
AGR	r	0.130	0.064	0.109	0.207*	0.114
	p	0.091	0.255	0.132	0.016	0.121
CON	r	0.228**	0.121	0.220*	0.150	0.127
	p	0.009	0.102	0.011	0.062	0.096
HLoC-In	r	0.226**	0.050	0.211*	0.168	0.171*
	p	0.010	0.303	0.015	0.041	0.039
HLoC-PO	r	0.272^∗∗^	0.044	0.295**	0.285**	0.276**
	p	0.002	0.326	0.001	0.001	0.002
HLoC-Ch	r	0.172*	0.010	0.109	0.138	0.215*
	p	0.038	0.459	0.133	0.079	0.013
GSE	r	0.170*	0.078	0.129	0.280**	0.124
	p	0.038	0.214	0.094	0.002	0.102
GHQ-28 Som	r	0.104	0.139	0.066	0.058	0.021
	p	0.287	0.154	0.499	0.551	0.829
GHQ-28 Anx	r	0.009	0.076	0.082	0.038	–0.194*
	p	0.926	0.434	0.400	0.701	0.046
GHQ-28 Soc	r	0.022	0.130	–0.111	0.045	0.005
	p	0.825	0.183	0.257	0.649	0.957
GHQ-28 Dep	r	–0.026	–0.051	–0.088	0.006	0.061
	p	0.787	0.603	0.368	0.955	0.532
GHQ-28 Diss	r	0.041	0.106	0.013	0.044	–0.053
	p	0.678	0.277	0.897	0.649	0.585
Age	r	0.185	0.170	0.155	0.114	0.115
	p	0.056	0.080	0.110	0.214	0.237
Age at the time of HTx	r	0.217*	0.206*	0.195*	0.148	0.128
	p	0.025	0.034	0.045	0.129	0.188
Number of years after HTx	r	–0.158	–0.120	–0.127	–0.158	–0.125
	p	0.103	0.218	0.192	0.105	0.199

*p < 0.05; **p < 0.01.

*HTx – heart transplantation; HBI – heath behavior index; HDH – healthy dietary habits; PB – prophylaxis behavior; PMA – positive mental attitude; EHP – everyday healthy practices; NEU – Neuroticism; EXT – Extraversion; OPN – Openness to experience; AGR – Agreeableness. CON – Conscientiousness. HLoC-In – Health Locus of Control – Internal; HLoC-PO – Health Locus of Control – Powerful Others; HLoC-Ch – Health Locus of Control – Chance; GSE – Generalized Self-Efficacy; GHQ-28 Som – Somatic Symptoms; GHQ-28 Anx – Anxiety with Insomnia; GHQ-28 Soc – Social Disfunction; GHQ-28 Dep – Depression; GHQ-28 Diss – Overall Distress.*

Based on the above results, it can be concluded that among heart transplant patients, the intensity of health behaviors is related to the belief about the ability to successfully cope with difficulties along with the health locus of control, that is, the belief in both one’s own influence on health or the influence of others (e.g., medical personnel) or random factors. Almost all the above-mentioned relationships are positive, meaning that as the level of a trait increases, so does the level of overall health care among heart transplant patients. The one exception regards the relationship between everyday healthy practices and openness to experience, which suggests that in our study group the lower level of openness, the higher level of health practices related to a healthy proportion of physical effort and sleep or rest.

### Age, Education, Distress, and Health Behaviors

We have analyzed the level of overall distress in the study group, as well as the level of reported types of difficulties regarding somatic symptoms, anxiety with insomnia, social dysfunctions, and depression. Comparing those results with norms for adults, proposed by the authors of GHQ-28, we have found that in transplant recipients 41 patients (38.3% of the group) reported low levels of overall distress. 40 participants (37.4% of the group) declared average, and 26 participants (24.3% of the study group) – high level of overall distress (Goldberg et al., 2001). The results of the correlation analyses suggest that among the subjects, the level of anxiety with insomnia is significantly associated with the intensity of daily healthy practices (*r* = -0.19, *p* < 0.05). This correlation has weak power (less than 0.3) and is negative, i.e., among heart transplant patients, high levels of anxiety cooccur with lower intensity of practices regarding adequate sleep and rest (Table 5).

Moreover, the analyses indicate positive relationships (with weak power) between age at the time of heart transplantation and overall health behavior index, dietary habits, and preventive behaviors (*r* = 0.22, *p* < 0.05; *r* = 0.21, *p* < 0.05; *r* = 19, *p* < 0.05, respectively). There was also a significant difference between the intensity of positive mental attitude in the groups with lower and higher levels of education (*U* = 914.50; *p* < 0.05) (Table 6). The results suggest that older heart transplant recipients are more likely to engage in health behaviors (in general) and are characterized by greater commitment to dietary compliance and preventive behaviors. At the same time, those with lower levels of education show higher levels of mental health concern compared to those with higher levels of education.

**TABLE 6:** The difference in health behavior between patients with lower (primary/occupational) and higher (secondary/higher) levels of education.

	HBI	HDH	PB	PMA	EHP
Mann-Wilcoxon U	1082.50	1267.00	1135.50	914.50	1016.00
Significance (two-tailed)	0.21	0.99	0.38	0.02*	0.09

*p < 0.05.

*HBI – heath behavior index; HDH – healthy dietary habits; PB – prophylaxis behavior; PMA – positive mental attitude; EHP – everyday healthy practices.*

### Personality Traits as Predictors of Health Behavior

Integration of the analysis was obtained by examining regression models for predicting health behaviors using significant personality coefficients of the overall HBI only, since it had, as only dependent variable, normal distribution of data. The psychological variables: Conscientiousness, Internal Locus of Control, External Locus of Control (Powerful Others), External Locus of Control (Chance) and Generalized Self-Efficacy were introduced to the regression model in above-mentioned order. Among tested models, the best prediction has the one with 3 factors: conscientiousness and health locus of control (Internal and Powerful Others) (Table 7). The regression model has shown goodness-of-fit at the acceptable level [*F*(3;103) = 5,747; *p* < 0,001] and predicts 14.3% of HBI variation in the studied group (*R*^2^ = 0,143). According to this model the strongest predictor of the engagement in health behavior is the level of external locus of control (Powerful Others) (β = 0,251; *p* < 0,05).

**TABLE 7:** The linear multinomial regression model for predicting overall health behavior index (HBI) using personality coefficients.

Model		Unstandardized coefficients		Standardized coefficients	*t*	*p*
		*B*	Std. Error	Beta		
1	(Constant)	76.489	6.946		11.012	0.000
	CON	0.503	0.210	0.228	2.397	0.018
2	(Constant)	68.253	7.937		8.599	0.000
	CON	0.434	0.210	0.196	2.067	0.041
	HLoC-In	0.426	0.208	0.194	2.048	0.043
3	(Constant)	64.413	7.876		8.179	0.000
	CON	0.441	0.204	0.200	2.159	0.033
	HLoC-In	0.227	0.217	0.104	1.047	0.297
	HLoC-PO	6.061	2.362	0.251	2.567	0.012

*CON – Conscientiousness. HLoC-In – Health Locus of Control – Internal; HLoC-PO – Health Locus of Control – Powerful Others.*

## Discussion

Insufficient engagement in health behaviors and unsatisfactory level of medication adherence are significant problems after vascular organ transplantation. Since the transplantation is a form of treatment in which outcomes are closely related to compliance, adaptation to the patient role has been the focus of many studies. They attempt to seek answers to questions about the extent of cooperation of organ transplant recipients and examine factors influencing treatment adaptation and effective interventions regarding motivation in this group of patients. The main objectives of this study were to verify the importance of subjective characteristics of heart transplant patients on their health behavior.

The results of the analyses indicate that vast majority (71%) of the study participants report a high level of engagement in various forms of health behaviors. Because daily health habits are an adequate indicator of adherence, the data from the present study suggest that approximately 30% of individuals in the study population exhibit difficulties in cooperating with the treatment process after transplantation (Siwińska et al., 2011). These findings are consistent with previous reports that 20-50% of transplant patients reveal problems with adherence to various categories of medical recommendations (Grady and Jalowiec, 1995; Laederach-Hofmann and Bunzel, 2000; Dew et al., 2007). The study group placed significantly less importance on adherence to dietary recommendations than on other types of health-relevant behaviors. No significant empirical rationale was found for determining whether preventive behaviors (such as participation in checkups or outpatient visits) were significantly higher compared to other types of health behaviors. These findings correspond with studies of other transplant patient groups, in which the highest rates of cooperation are being observed in relation to medication adherence (especially to the immunosuppressive treatment) and the lowest – to dietary recommendations (Hreńczuk et al., 2018).

The analyses allowed us to identify variables that may increase the likelihood of engaging in health-enhancing behaviors. Of the possible correlates of health behaviors examined, both demographic and psychological factors proved to be important for adaptation to treatment in the study group. According to the results, older age at the time of surgery favors the adoption of favorable behaviors in the subsequent course of treatment, including attention to proper eating habits and proper contact with health care. These relationships are also reflected in other studies in the field of transplantation (Kotarska et al., 2015). Young age at the time of transplantation may be an important risk factor – Aujnarain et al. (2017) pointed out that younger people reveal significantly more difficulties in compliance after organ transplantation (Aujnarain et al., 2017). Hence, the necessity for exceptional care given to younger individuals, especially those who underwent transplantation in childhood and those who are in transition to an adult center. Only adult patients participated in the current study, but a similar direction of relationship as in studies of child and adolescent populations suggests that those who experienced transplantation at a younger age are less likely to care for their own health and should receive adequate care from the transplant team.

The level of education was also found to be a significant factor in the study group; however, the direction of this relation was different from the cited research results and our assumptions. Among the study participants, those with lower education were the group taking more care of positive psychological attitude (by avoiding experiencing strong emotions and using strategies to reduce stress levels). The literature suggests that a lower level of general knowledge and, as a result, a low level of health literacy, is a risk factor for incomplete adherence, poorer quality of life, and adverse events during treatment (White and Gallagher, 2010; Cajita et al., 2017). The results of these studies were not confirmed by the results of the current analyses. However, these specific correlations in the study group seem to reflect theoretical relationships described, for example, in Susan Miller’s concept of coping with stress. According to this theory, it is possible that with a higher level of health knowledge, the level of anxiety related to the possible consequences of the current situation may increase. While greater knowledge may positively influence medication adherence and other preventive behaviors, it has an adverse effect on psychological well-being by increasing levels of health anxiety. At the same time, findings from other populations indicate a tendency for individuals with lower levels of education to have avoidance-focused rather than task-focused coping. According to these hypotheses, in individuals with lower levels of education, we are more likely to expect behaviors that bring about a reduction in the level of psychological discomfort than behaviors aimed at changing the stressful situation (here, adherence). This hypothesis was only partially verified in the process of analyzing the results and may be an interesting development of the research problem presented in this paper.

The results of the current study also did not confirm the relationship described in other studies, in which one of the factors that potentially influenced adherence to recommendations was time since surgery. In a study by Germani et al. (2011), those with longer survival time since surgery revealed greater severity of behaviors indicative of insufficient cooperation, whereas in the current study this relationship did not reach the level of statistical significance (Germani et al., 2011).

Another area explored in the present study was the relationship of reported psychological difficulties to health behaviors. Among the study participants, 24.3% revealed high level of distress, which confirms findings from other studies (Kugler et al., 2014). Despite numerous reports on the relationship between the presence of psychopathological symptoms and health care, the results obtained in the present study do not support these assumptions. In the study group, the level of reported psychological difficulties was not significantly related to the engagement in health behaviors. The exception was the relationship between levels of anxiety and insomnia and daily healthy practices. This form of health behavior is related specifically to adherence to the proper balance between exercise/activity and rest and sleep hygiene. Hence, the association between reported insufficient rest in individuals complaining of sleep disorders seems reasonable. At the same time, this highlights a critical area of functioning in post-transplant patients that has not yet been explored in the literature. Sleep as a basic physiological activity ensuring sympathetic-parasympathetic balance is a major area of quality of life and proper functioning. Sleep hygiene difficulties reported by study participants and their relationship to levels of psychopathological symptoms may warrant further exploration.

### The Influence of Subjective Characteristics on the Level of Health Behaviors

The main purpose of the research conducted in this study was to determine the relationship between health habits and personality characteristics, and the results provided interesting conclusions. Among the analyzed subjective characteristics, only generalized self-efficacy was proven to be significant predictor of health-enhancing behaviors. The results suggest that individuals with an elevated level of belief in his or her capacity to execute behaviors necessary to produce specific performance attainments will be significantly more engaged in various forms of health behaviors. These results confirm the importance attributed to these traits in cooperation after transplantation and in other clinical contexts. In Życińska’s (2017) study, the sense of self-efficacy and internal locus of health control were also significant factors influencing adaptation, but in her study population (patients with diabetes and acute coronary syndrome) they were predictors of emotional adaptation and well-being of patients rather than variables influencing health behaviors (Życińska, 2017). The belief that one can influence one’s own health, the attaching of importance to the guidance and actions of physicians along with a stable tendency to act in an orderly manner are, as hypothesized, important resources in the process of adaptation to treatment after heart transplantation. These findings support previous analyses on the protective effects of personality factors in the health behavior domain (Dobbels et al., 2004; Telles-Correia et al., 2009; Kronish and Ye, 2013; Silva et al., 2016; Kohli, 2017). These results interpreted in pathogenetic terms, suggest that a propensity for impulsive and chaotic behavior and a belief in negligible or insignificant self-influence (or the actions of health care providers) on health may be risk factors for difficulties in adherence. The higher risk of unhealthy behaviors can also occur in individuals with the belief about not being able to succeed in specific situations or accomplish a task, such as following medical recommendations. For the above reasons, these results can be described as an indication to deepen the psychological assessment of transplant patients in the area of attributional beliefs and personality characteristics. Regarding the meta-resources, it can be stated that the results obtained support, in part, the concept of mental toughness, indicating the importance of its pillars, namely, self-efficacy, internal locus of control and conscientiousness (described as “confidence,” “control,” and “commitment” in this theory). Since there are few studies on this construct, verifying the importance of mental toughness in the process of adaptation to treatment after organ transplantation could be an interesting development of the current research problem.

### Limitations of the Study

The current research project was conducted with methodological and substantive assumptions that were intended to structure the argument and methodology of this study. At the same time, however, they may represent some limitations of this study. The first limitation was the lack of a comparison group in the implemented research model. Due to the assumption of the exploratory nature of the study, a group that could serve as an interpretative background for the population under study was dropped. The analysis of studied variables in other populations, such as patients after different organ transplants, could be an interesting development of the described research problem and allow the generalization of the study results to other clinical groups. Another limitation was the focus on subjective psychological resources. As the literature suggests, correlates of health behaviors may also be interpersonal or organizational in nature, resulting in these areas being explored in other adherence research. Another limitation of the study derives from the possible source of response bias related to patients’ need to show themselves as cooperative and behaving according to medical recommendations. We have tried to address that concern providing patients with support during completing questionnaires and acceptance of their difficulties with adherence in the clinical setting. Even though the questionnaire set was covering a broad range of variables and consisted of multiple items, majority of the patients manifested engagement in the study and appreciated the need of understanding the psychological factors which could enhance their compliance. The sheer number of participants in the study is also a certain limitation. As suggested in recent years, in scientific projects in the field of health psychology, the aim should be to increase the size of the study population to achieve statistically and clinically significant results and thus, interpretable in broader contexts (Jewett et al., 2010). At the same time, however, the current study was able to obtain data from over 33% of the current group of transplant patients under the care of the Institute of Cardiology in Warsaw, which can be considered a representative sample for this center. Additionally, results corresponding with reports from other centers indicate the existence of similar relationships in groups of transplant patients after transplantation of other organs and from other medical centers.

## Conclusion

Considering the obtained results, it seems reasonable to conclude that most heart transplant patients are characterized by high levels of health behaviors. Among the various forms of health-relevant behaviors, heart transplant patients engage significantly less frequently in behaviors related to a healthy diet than in preventive behaviors, attention to a positive mental attitude, or daily health practices. Among all subjective resources examined, the best predictors of caring about health are health locus of control, and level of conscientiousness. The results of the analyzes also indicate other relationships with respect to specific forms of health behaviors.

## Data Availability Statement

The raw data supporting the conclusions of this article will be made available by the authors, without undue reservation.

## Ethics Statement

The studies involving human participants were reviewed and approved by Bioethics Committee of the Institute of Cardiology. The patients/participants provided their written informed consent to participate in this study.

## Author Contributions

AM, AK, MS-M, TZ, and RP contributed to conception and design of the study. AM and GJ-W organized the database. AM and AK performed the statistical analysis and wrote the first draft of the manuscript. All authors contributed to manuscript revision, read, and approved the submitted version.

## Conflict of Interest

The authors declare that the research was conducted in the absence of any commercial or financial relationships that could be construed as a potential conflict of interest.

## Publisher’s Note

All claims expressed in this article are solely those of the authors and do not necessarily represent those of their affiliated organizations, or those of the publisher, the editors and the reviewers. Any product that may be evaluated in this article, or claim that may be made by its manufacturer, is not guaranteed or endorsed by the publisher.
